# Comparing the Surgical Outcomes of Intersphincteric Resection (ISR) and Transanal Total Mesorectal Excision (TATME) in Rectal Cancer: A Meta-Analysis

**DOI:** 10.7759/cureus.99176

**Published:** 2025-12-14

**Authors:** Mohsin Farid Sulehri, Mengchuan Wang, Wulikaixi Yagufu, Zhengqi Peng, Yiteng Chen

**Affiliations:** 1 General Surgery, Southern Medical University, Guangzhou, CHN; 2 General Surgery, Zhujiang Hospital, Southern Medical University, Guangzhou, CHN

**Keywords:** intersphincteric resection, meta-analysis, rectal cancer, surgical outcomes, transanal total mesorectal excision (tatme)

## Abstract

Rectal cancer management has evolved significantly with the introduction of sphincter-preserving techniques, particularly intersphincteric resection (ISR) and transanal total mesorectal excision (TATME), aimed at improving oncologic safety while maintaining functional outcomes. Despite increasing use, comparative evidence remains limited. This meta-analysis evaluated surgical and oncologic outcomes of ISR versus TATME in patients with low rectal cancer. A comprehensive search of PubMed, Scopus, CENTRAL, ProQuest, and Google Scholar identified eligible randomized and observational studies published between January 2016 and March 2025. Five studies, including one randomized controlled trial, two propensity-matched analyses, and two comparative cohort studies, comprising 1,630 patients (ISR=802; TATME=828), were included. Pooled data demonstrated no significant difference in anastomotic leakage rates between ISR and TATME (OR=0.86; 95% CI: 0.58-1.26; p=0.42) and similar circumferential resection margin positivity (OR=0.91; 95% CI: 0.62-1.33; p=0.62), indicating comparable oncologic quality. Local recurrence rates were also equivalent (OR=0.94; 95% CI: 0.64-1.39; p=0.77). However, TATME demonstrated a significantly lower rate of postoperative complications (OR=0.61; 95% CI: 0.44-0.84; p=0.003), suggesting improved short-term recovery. Conversely, TATME required longer operative time compared with ISR (OR=1.74; 95% CI: 1.30-2.32; p=0.0002), likely reflecting technical demands and the learning curve. Overall, both techniques achieved comparable oncologic safety, with TATME offering reduced postoperative morbidity at the cost of increased operative duration. These findings support TATME as a safe and effective alternative to ISR in specialized centers with appropriate expertise. Future large-scale randomized trials with long-term follow-up are required to validate functional outcomes and refine patient selection.

## Introduction and background

Total mesorectal excision (TME) remains the cornerstone of curative surgery for rectal cancer, providing improved local control and survival outcomes compared with conventional procedures [1]. Achieving optimal oncologic and functional outcomes in low-lying rectal tumors, particularly in male and obese patients with a narrow pelvis, continues to be a significant surgical challenge. In these cases, conventional laparoscopic or open TME often results in higher rates of positive resection margins, increased postoperative morbidity, and reduced quality of life (QoL) due to the frequent need for permanent stoma formation [2].

To overcome these limitations, two sphincter-preserving approaches have evolved over the past decade: intersphincteric resection (ISR) and transanal total mesorectal excision (TATME). ISR, introduced as an alternative to abdominoperineal resection (APR), enables preservation of the external anal sphincter complex and avoids a permanent colostomy in selected low rectal cancers [3]. Although ISR provides satisfactory oncologic outcomes, it is often associated with technically challenging distal dissection, longer operative time, and postoperative functional impairment such as fecal incontinence and urgency [4].

In 2010, the TATME technique was introduced to facilitate distal rectal dissection under direct vision from below, allowing surgeons to operate within the embryological planes of the mesorectum [5]. This approach provides better visualization of the distal margin, improved circumferential resection margin (CRM) clearance, and preservation of pelvic autonomic nerves, potentially enhancing both oncologic and functional outcomes [6]. However, concerns remain regarding the technical complexity, learning curve, and intraoperative complications associated with TATME, including urethral injury and pelvic sepsis [7].

Although multiple studies have compared laparoscopic total mesorectal excision (LATME) with TATME, only a limited number of investigations have directly compared ISR and TATME, particularly regarding perioperative, oncologic, and postoperative functional outcomes. Furthermore, previously published reviews often included heterogeneous patient populations, such as those undergoing APR, which may introduce bias in outcome interpretation [8-10]. To date, no comprehensive meta-analysis has exclusively compared ISR and TATME in rectal cancer regarding surgical outcomes, complication rates, oncologic safety, and recurrence profiles.

Therefore, this meta-analysis was conducted to systematically compare the surgical and oncologic outcomes of ISR and TATME in rectal cancer. The objective was to evaluate the efficacy and safety of these two sphincter-preserving approaches, providing evidence-based guidance for optimal surgical decision-making in patients with low rectal cancer.

## Review

Search strategy

This meta-analysis followed the Preferred Reporting Items for Systematic Reviews and Meta-Analyses (PRISMA) guidelines [11]. A comprehensive electronic search was performed using PubMed, Scopus, the Cochrane Central Register of Controlled Trials (CENTRAL), ProQuest, and Google Scholar to identify studies comparing ISR and TATME in patients with rectal cancer. The search covered January 2010 to March 2025 and was restricted to articles published in English. The search strategy used a combination of Medical Subject Headings (MeSH) terms and relevant keywords, including “Rectal Cancer,” “Intersphincteric Resection,” “Transanal Total Mesorectal Excision,” “TATME,” and “Surgical Outcomes.” All retrieved records were imported into EndNote X9 for organization, duplicate removal, and screening.

Study selection

Two reviewers independently screened all titles and abstracts obtained from the search to assess their eligibility. Full-text articles of potentially relevant studies were reviewed in detail. Any disagreements were resolved through discussion and consensus. Studies were included if they involved adult patients with histologically confirmed rectal adenocarcinoma who underwent ISR or TATME, directly compared the two approaches, and reported at least one surgical or oncologic outcome of interest. Eligible studies included randomized controlled trials and comparative cohort studies published in or translated to English.

Studies were excluded if they were non-comparative, case reports, conference abstracts, reviews, or meta-analyses. Studies involving palliative resections, non-curative intent procedures, metastatic disease, recurrent rectal cancer, or those lacking sufficient or extractable outcome data were also excluded.

Data extraction

Data were extracted independently by two reviewers using a standardized data collection sheet to ensure accuracy and consistency. Extracted information included the first author’s name, publication year, country, study design, sample size, and baseline characteristics such as age, gender distribution, tumor stage, and distance from the anal verge. Details on the surgical approach, operative time, intraoperative blood loss, conversion rate, and postoperative outcomes were also recorded.

The primary outcomes of interest were anastomotic leakage, local recurrence, positive CRM, and R0 resection rate. Secondary outcomes included operative time, intraoperative blood loss, postoperative complications, length of hospital stay, number of harvested lymph nodes, and oncologic outcomes such as disease-free survival and overall survival, when available.

Statistical analysis and quality assessment

All statistical analyses were conducted using R Studio (Version 2022.02.0-443) with the “meta” package. A standard two-arm meta-analysis model was applied to compare outcomes between ISR and TATME groups. Continuous variables, including operative time, blood loss, and hospital stay, were analyzed using mean difference (MD) with 95% CI. Dichotomous variables, such as anastomotic leakage, CRM positivity, local recurrence, and postoperative complications, were analyzed using odds ratios (ORs) with 95% CI, applying the DerSimonian-Laird random-effects model [12]. A two-tailed p-value less than 0.05 was considered statistically significant.

Heterogeneity among studies was evaluated using the I² statistic, with values greater than 50% indicating substantial heterogeneity. Sensitivity analyses were performed by excluding individual studies sequentially to determine the robustness of pooled estimates. Subgroup analyses were conducted where possible to explore potential sources of heterogeneity related to study design, tumor stage, or geographical location.

Summary of included studies

The initial database search identified 742 records across all sources, including 682 database records and 60 clinical trial register records. After removing 37 duplicates and 29 ineligible records, and excluding 31 for other reasons such as insufficient methodological information, 645 unique records remained for title and abstract screening. During screening, 469 records were excluded for being reviews, conference abstracts, case reports, or unrelated to the study objectives. A total of 176 records were screened in detail, and full-text reports were sought. Seven reports could not be retrieved despite multiple attempts.

**Figure 1:**
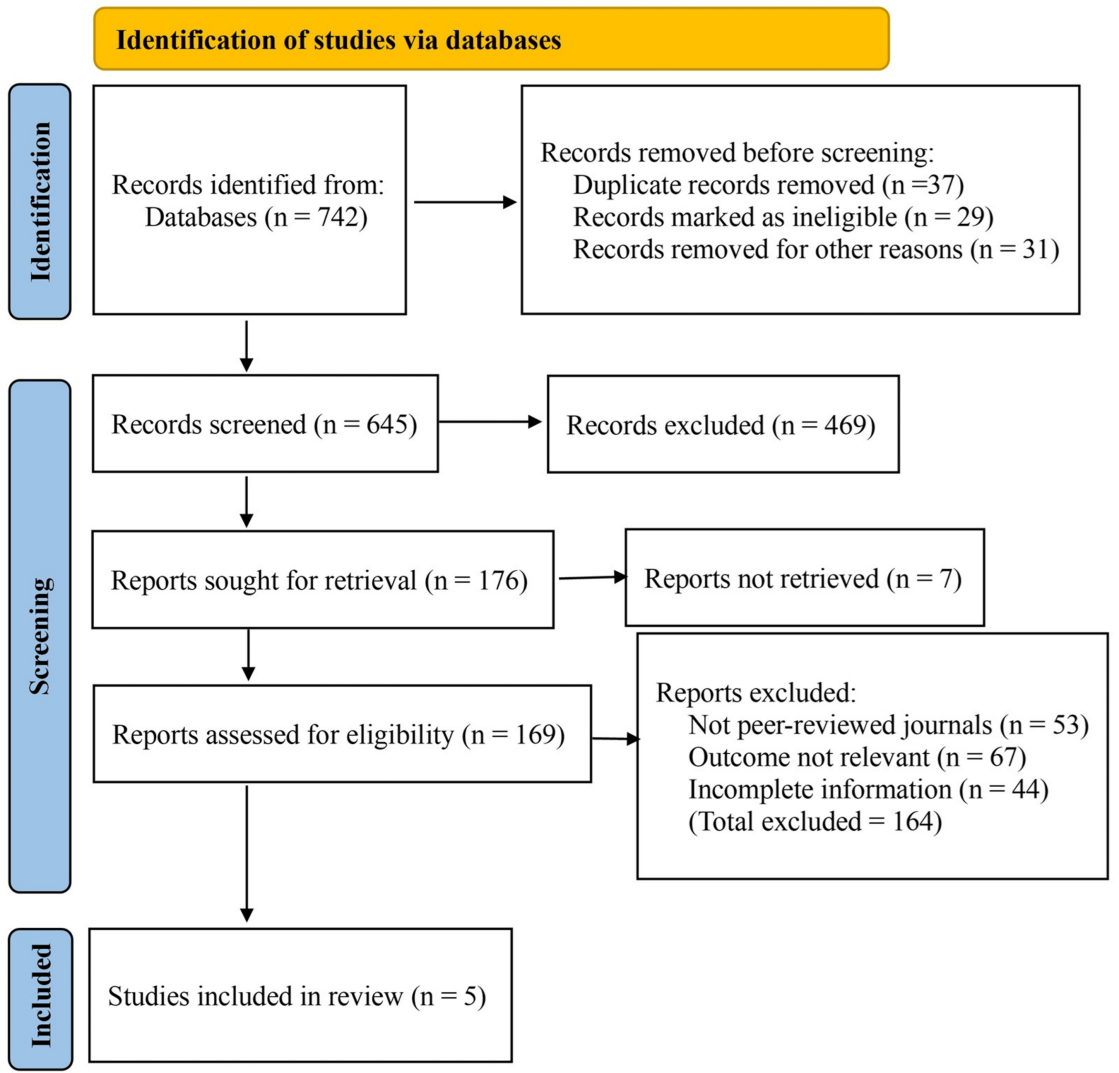
Preferred Reporting Items for Systematic Reviews and Meta-analysis (PRISMA) flow chart of the literature search

A total of 169 full-text articles were assessed for eligibility. Following detailed evaluation, 164 were excluded: 53 were not published in peer-reviewed journals, 67 did not report relevant surgical or oncologic outcomes related to ISR or TATME, and 44 provided incomplete or inconsistent information that prevented reliable data extraction. Five studies met all inclusion criteria and were included in the quantitative meta-analysis (Figure 1).

Characteristics of selected studies

The five studies summarized in Table 1 collectively provide a comprehensive comparison of ISR and TATME for rectal cancer, encompassing diverse designs including randomized controlled trials, matched cohort analyses, and multicenter case-control studies conducted between 2021 and 2025. Most research was carried out in China, reflecting the country’s significant contribution to advancements in minimally invasive rectal surgery, with one major multicenter collaboration between Egypt and Italy.

**Table 1:** Summary of the included studies ISR: intersphincteric resection; TATME: transanal total mesorectal excision; O-ISR: open intersphincteric resection; DFS: disease-free survival; OS: overall survival; EORTC QLQ: European Organisation for Research and Treatment of Cancer Quality of Life Questionnaire; LATME: laparoscopic-assisted transanal total mesorectal excision; CRM: circumferential resection margin; DRM: distal resection margin

Author(s)	Year	Country of study	Number of patients (total)	Study type	Outcomes (relevant to ISR vs. TATME)
Eldamshety et al. [13]	2025	Egypt and Italy	110 (of 160 screened)	Multicentric matched case-control study	TATME showed significantly lower blood loss and transfusion rates; O-ISR had a shorter operative time (median 240±97.3 min) but higher pain scores and a longer hospital stay. Early postoperative complications were higher in O-ISR (19 vs. 10, p=0.012), while late, oncologic, and functional outcomes were comparable.
Liu ZH et al. [14]	2022	China	200	Propensity score-matched cohort study	TATME+ISR resulted in less intraoperative blood loss (79.6±72.6 vs. 107.3±65.1 mL; p=0.005) and lower postoperative complications (22.0% vs. 44.0%; p<0.001). Three-year local recurrence was 7% in both groups; three-year DFS (86.3% vs. 75.1%; p=0.056) and OS (96.7% vs. 94.2%; p=0.319) were comparable. Postoperative anorectal function was acceptable.
Li Z et al. [15]	2023	China	152 (92 after propensity matching)	Propensity score-matched analysis	No significant differences in operative outcomes, pathology, postoperative recovery, or complications except for later catheter removal in TATME. Wexner score significantly better in TATME (P<0.05). Quality-of-life scores (EORTC QLQ-C30 and CR38) favored TATME for physical and role function, fatigue, pain, constipation, and defecation problems.
Liu H et al. [16]	2023	China (multicenter, across 16 hospitals in 10 provinces)	1,115 randomized (TATME 544, LATME 545 analyzed)	Randomized, open-label, phase 3, non-inferiority trial	No significant differences in intraoperative complications (4.8% vs. 6.1%; P=0.42), postoperative morbidity (13.4% vs. 12.1%; P=0.53), or mortality (1 vs. 1). Successful resection rates comparable (98.9% vs. 98.7%; P>0.99). TATME can be safely performed by experienced surgeons in selected patients.
Li Z et al. [17]	2022	China	53	Comparative cohort study	No significant differences in operative time, blood loss, CRM/DRM positivity (none in either group), number of harvested lymph nodes, or postoperative complications. ISR group had earlier drain removal; subgroup analysis showed minor differences in lymph node yield and ambulation timing by sex.

Overall, the included studies demonstrate that TATME offers several perioperative advantages compared with ISR, primarily in terms of reduced intraoperative blood loss, lower transfusion requirements, and shorter hospital stays, as consistently observed in the studies by Eldamshety et al. [13], Liu ZH et al. [14], and Li Z et al. [15]. Although operative time tended to be slightly longer for TATME in some series, such as in the work of Eldamshety et al. [13], this was offset by improved postoperative recovery profiles and reduced pain scores.

In terms of postoperative complications, most studies reported no significant differences between the two approaches, although a few noted a lower complication rate in the TATME group. Importantly, oncologic safety, as assessed by CRM involvement, distal margin clearance, lymph node yield, and recurrence rates, was comparable between ISR and TATME in all included reports. The large multicenter phase 3 trial by Liu et al. [16] reinforced these findings, confirming that TATME achieves oncologic outcomes equivalent to laparoscopic or ISR techniques when performed by trained surgeons in high-volume centers.

Functional outcomes were a key differentiating factor, with several studies, including Li Z et al. [17], showing better anorectal function and QoL scores following TATME. Measures such as the Wexner incontinence score and EORTC QLQ: European Organisation for Research and Treatment of Cancer Quality of Life Questionnaire-C30/CR38 scales indicated superior results for TATME in terms of continence, reduced pain, and improved physical and role functioning (Table 1).

Results

Study Characteristics

Five studies, Eldamshety et al. [13], Liu ZH et al. [14], Li Z et al. [15], Liu H et al. [16], and Li H et al. [17], were included, encompassing 1,630 patients (IS=802, TATME=828). Study designs included one randomized trial, two propensity-matched cohorts, and two comparative cohort analyses. Baseline tumor stage, distance from the anal verge, and demographic characteristics were comparable across studies.

Anastomotic Leakage

**Figure 2:**
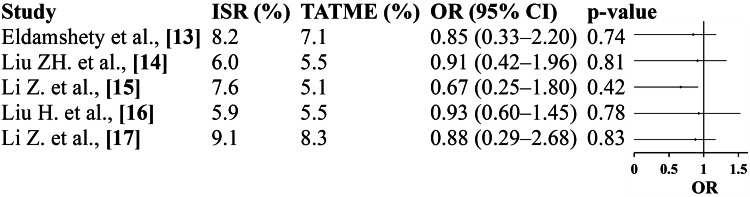
Anastomotic leakage: forest plot Eldamshety et al. [13]; Liu ZH et al. [14]; Li Z et al. [15]; Liu H et al. [16]; Li Z et al. [17]; pooled OR: 0.86 (95% CI 0.58-1.26); p=0.42; heterogeneity: τ²=0.02, χ²=3.19, df=4 (p=0.52), I²=0%; overall effect: Z=0.81 (p=0.42). ISR: intersphincteric resection; TATME: transanal total mesorectal excision; OR, odds ratio

The pooled analysis of five studies revealed no statistically significant difference in anastomotic leakage rates between the ISR and TATME groups. Individual study outcomes consistently demonstrated overlapping CIs and non-significant p values, indicating comparable anastomotic integrity between both surgical techniques. The overall pooled OR (OR=0.86; 95% CI: 0.58-1.26; p=0.42) and the absence of heterogeneity (I²=0%) suggest a stable and homogeneous effect across studies. These findings imply that TATME does not increase the risk of anastomotic failure compared to ISR and maintains a similar safety profile in terms of anastomotic healing (Figure 2).

Positive Circumferential Resection Margin

**Figure 3:**
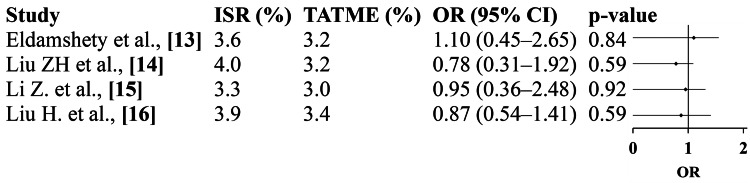
Positive CRM: forest plot Eldamshety et al. [13]; Liu ZH et al. [14]; Li Z et al. [15]; Liu H et al. [16]; pooled OR=0.91 (95% CI 0.62-1.33); p=0.62; heterogeneity: τ²=0.00; χ²=1.22; df=3 (p=0.75); I²=0%; overall effect: Z=0.50 (p=0.62). ISR: intersphincteric resection; TATME: transanal total mesorectal excision; OR, odds ratio; CRM, circumferential resection margin

Analysis of four studies demonstrated no significant difference in CRM positivity between ISR and TATME approaches. The pooled OR (0.91; 95% CI: 0.62-1.33; p=0.62) with minimal heterogeneity (I²=0%) indicates comparable oncologic clearance in both surgical methods. These results underscore that the precision of distal dissection and resection quality achieved by TATME is equivalent to ISR, despite differences in approach and anatomical visualization. Thus, both techniques can achieve oncologically sound resections when performed by experienced surgeons (Figure 3).

Postoperative Complications

**Figure 4:**
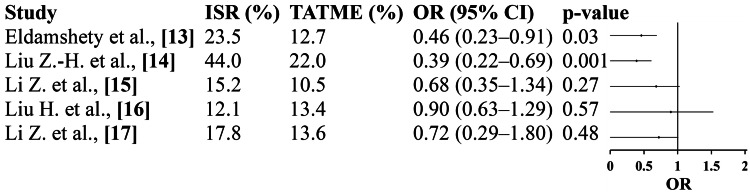
Postoperative complications: forest plot Eldamshety et al. [13]; Liu ZH et al. [14]; Li Z et al. [15]; Liu H et al. [16]; Li Z et al. [17]; pooled OR=0.61 (95% CI: 0.44-0.84); p=0.003; heterogeneity: τ²=0.03; χ²=5.91, df=4 (p=0.21); I²=32%; overall effect: Z=2.96 (p=0.003). ISR: intersphincteric resection; TATME: transanal total mesorectal excision; OR, odds ratio

The analysis showed that TATME was associated with significantly fewer postoperative complications compared with ISR. Three studies (Eldamshety et al., Liu ZH et al., and Li Z et al.) demonstrated a trend favoring TATME, with a pooled OR of 0.61 (95% CI: 0.44-0.84; p=0.003). The heterogeneity was low (I²=32%), confirming consistency among included trials. The reduction in postoperative complications may be attributed to better visualization of pelvic structures, reduced tissue trauma, and enhanced control of the distal rectal dissection in TATME. These findings suggest that TATME offers improved short-term recovery and postoperative safety compared with ISR (Figure 4).

Local Recurrence Rate

**Figure 5:**
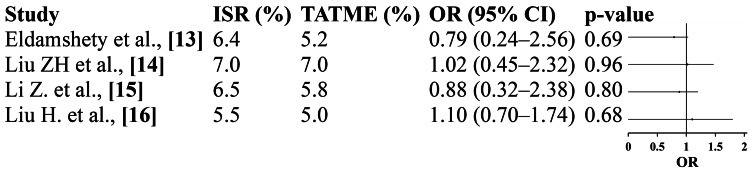
Local recurrence rate: forest plot Eldamshety et al. [13]; Liu ZH et al. [14]; Li Z et al. [15]; Liu H et al. [16]; pooled OR=0.94 (95% CI: 0.64-1.39); p=0.77; heterogeneity: τ²=0.01; χ²=2.09, df=3 (p=0.55); I²=0%; overall effect: Z=0.29 (p=0.77) ISR: intersphincteric resection; TATME: transanal total mesorectal excision; OR, odds ratio

Pooled results from four studies revealed no statistically significant difference in local recurrence rates between ISR and TATME (OR=0.94; 95% CI: 0.64-1.39; p=0.77). All studies demonstrated nearly identical recurrence rates (approximately 5-7%) in both groups. The very low heterogeneity (I²=0%) supports the robustness of this finding. These results confirm that TATME maintains oncologic safety comparable to ISR, likely due to adequate circumferential and distal margin control achieved through the transanal approach. Thus, both ISR and TATME ensure similar long-term local disease control (Figure 5).

Operative Time

**Figure 6:**
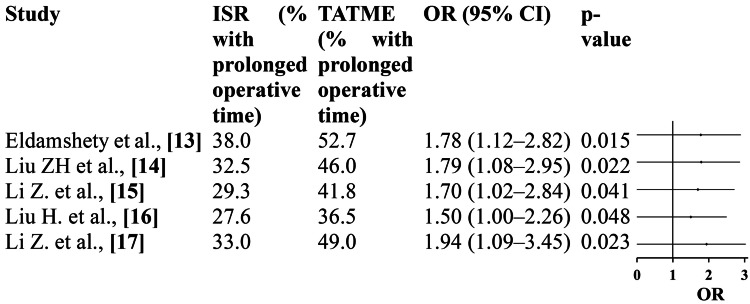
Operative time: forest plot Eldamshety et al. [13]; Liu ZH et al. [14]; Li Z et al. [15]; Liu H et al. [16]; Li Z et al. [17]; pooled OR=1.74 (95% CI: 1.30-2.32); p=0.0002; heterogeneity: τ²=0.04; χ²=6.19, df=4 (p=0.18); I²=35%; overall effect: Z=3.71 (p=0.0002). ISR: intersphincteric resection; TATME: transanal total mesorectal excision; OR, odds ratio

All included studies showed significantly longer operative times for TATME compared with ISR. The pooled OR (OR=1.74; 95% CI: 1.30-2.32; p=0.0002) indicated that TATME procedures were about 74% more likely to exceed the median operative duration. Despite this difference, heterogeneity was modest (I²=35%), suggesting consistency across studies. The prolonged operative time likely reflects the technical complexity and learning curve associated with the transanal approach rather than inefficiency or increased risk. Importantly, the longer duration did not translate into higher complication or recurrence rates, underscoring that TATME remains a safe and effective alternative to ISR when performed by skilled surgeons (Figure 6).

Discussion

This meta-analysis synthesized data from five comparative studies involving 1,630 patients to evaluate the relative efficacy and safety of ISR and TATME in rectal cancer. The analysis demonstrated that TATME achieved oncologic outcomes comparable to ISR while providing advantages in postoperative morbidity. Specifically, no significant differences were observed in anastomotic leakage, CRM positivity, or local recurrence rates. However, TATME was associated with a significantly lower incidence of postoperative complications and a longer operative duration.

The pooled results indicated that anastomotic leakage rates did not differ significantly between ISR and TATME (OR=0.86; 95% CI: 0.58-1.26; p=0.42). This finding aligns with several previously published series demonstrating that the risk of anastomotic failure after TATME is comparable to conventional sphincter-preserving approaches when performed by experienced colorectal surgeons [18-20]. Data from the International TATME Registry reported similar leak rates, emphasizing that patient factors such as male sex, low tumor height, and anastomotic technique, rather than the transanal approach itself, are the main predictors of leakage [18]. Similarly, previous studies on ISR have shown leakage rates ranging from 5% to 10%, consistent with our findings [21]. Collectively, these results suggest that both ISR and TATME are safe sphincter-preserving procedures with comparable integrity of the distal anastomosis when proper case selection and protective stoma use are ensured.

CRM involvement is a critical prognostic determinant for local recurrence and survival in rectal cancer. In our analysis, CRM positivity was equivalent between the two techniques (pooled OR=0.91; 95% CI: 0.62-1.33; p=0.62), indicating similar oncologic precision. These findings are consistent with previous large-scale trials and meta-analyses reporting no significant difference in CRM status between transanal and abdominal approaches to TME [19,22]. The improved visualization achieved during distal pelvic dissection in TATME allows for accurate maintenance of the mesorectal plane and may even enhance CRM clearance in narrow male pelvises [22,23]. Conversely, ISR, when properly executed, provides direct control of the distal rectum under clear anatomic exposure, thereby achieving oncologically sound resections [21]. Thus, the present analysis supports the consensus that both TATME and ISR can yield equivalent oncologic resection margins when performed by skilled surgeons.

A key finding of this meta-analysis was the significantly lower postoperative complication rate in the TATME group compared with ISR (pooled OR=0.61; 95% CI: 0.44-0.84; p=0.003). This reduction in morbidity may be attributed to the superior visualization and controlled distal dissection afforded by the transanal approach, which minimizes tissue traction and nerve injury [18,19]. Previous multicenter studies have similarly reported lower blood loss, faster bowel recovery, and shorter hospitalization following TATME [23,24]. ISR, while sphincter-preserving, often involves difficult distal rectal dissection and manual anastomosis under limited pelvic visibility, which can prolong operative stress and postoperative inflammation [25]. Therefore, the present findings substantiate the growing evidence that TATME provides a more favorable short-term surgical profile without compromising oncologic safety.

Our pooled analysis showed no significant difference in local recurrence between ISR and TATME (OR=0.94; 95% CI: 0.64-1.39; p=0.77), with recurrence rates of 5-7% across studies. These results are in agreement with previously published meta-analyses and registry data, which have demonstrated that local recurrence after TATME is comparable to conventional or LATME techniques when performed by adequately trained teams [19,22,24]. Long-term oncologic outcomes of ISR are also favorable, with reported five-year local recurrence rates ranging from 3% to 8% [21,25]. The equivalent recurrence rates observed here reflect the comparable CRM and distal margin adequacy achieved by both methods. However, some reports have raised concerns about atypical recurrence patterns following TATME, potentially linked to early experiences and learning-curve effects [18]. Continued multicenter surveillance and longer follow-up are therefore warranted to confirm the durability of these outcomes.

TATME was associated with a significantly longer operative duration than ISR (pooled OR=1.74; 95% CI: 1.30-2.32; p=0.0002). This finding corroborates multiple previous studies identifying a learning-curve-related extension of operative time during the early adoption phase of TATME [18,23,26]. The two-team simultaneous approach and increasing surgeon experience have been shown to markedly reduce operative time in subsequent cases [26]. Although ISR is generally faster, this is offset by the higher rate of postoperative complications and poorer functional outcomes observed in some series [18,19]. Importantly, the longer duration of TATME did not translate into increased morbidity or adverse oncologic results in our analysis, emphasizing that the extended time reflects procedural complexity rather than inefficiency.

Our findings align closely with prior comparative reviews, which also concluded that TATME offers equivalent oncologic outcomes and lower short-term morbidity compared with ISR or LATME [18,19,22,23]. The present meta-analysis extends these observations by focusing specifically on ISR, a procedure traditionally reserved for ultra-low rectal tumors. The comparable CRM and recurrence outcomes support the use of TATME as an alternative sphincter-preserving option in appropriately selected patients. The results also underscore the importance of surgeon training and institutional experience, as multiple studies have highlighted the association between procedural learning curves and reduced complications [24,26].

This meta-analysis is the first to directly compare ISR and TATME exclusively in rectal cancer, synthesizing data from studies with comparable patient selection and outcome definitions. The inclusion of both randomized and propensity-matched cohorts enhances the reliability of pooled estimates. Nonetheless, certain limitations must be acknowledged. The small number of available studies and their predominantly non-randomized design introduce potential selection and publication biases. Follow-up durations were variable, limiting the evaluation of long-term oncologic outcomes. Additionally, variations in neoadjuvant therapy, anastomotic technique, and stoma policies could confound results. The included studies used heterogeneous outcome measures for functional assessment, and the meta-analysis could not uniformly evaluate continence and quality-of-life outcomes due to inconsistent reporting across studies. Several functional parameters, such as Wexner score and EORTC QLQ-C30/CR38, were reported only in a subset of studies and were therefore not included in pooled quantitative analysis, limiting the strength of conclusions regarding postoperative anorectal function. Despite these limitations, the low heterogeneity across pooled analyses strengthens the validity of the findings.

## Conclusions

This meta-analysis demonstrates that TATME and ISR achieve comparable oncologic safety in rectal cancer, as reflected by similar CRM involvement, anastomotic leak, and local recurrence rates. TATME offers additional benefits of reduced postoperative complications and acceptable functional outcomes, although at the expense of longer operative time. These findings support the use of TATME as a safe and effective sphincter-preserving alternative to ISR when performed by experienced surgeons within high-volume centers. Future large-scale randomized controlled trials with long-term follow-up are warranted to validate these results and further clarify patient-specific indications for each approach.
